# Nutrition-Related Knowledge and Nutrition-Related Practice among Polish Adolescents—A Cross-Sectional Study

**DOI:** 10.3390/nu16111611

**Published:** 2024-05-25

**Authors:** Agata Wawrzyniak, Iwona Traczyk

**Affiliations:** 1 Department of Human Nutrition, Institute of Human Nutrition Sciences, Warsaw University of Life Sciences (SGGW-WULS), 02-787 Warsaw, Poland; 2 Department of Public Health, Faculty of Health Sciences, Medical University of Warsaw (MUW), 02-091 Warsaw, Poland; iwona.traczyk@wum.edu.pl

**Keywords:** nutritional recommendations, nutrition-related knowledge (NRK), nutrition-related practice (NRP), Polish adolescents

## Abstract

The aim of this study was to examine the association between nutrition-related knowledge (NRK) and nutrition-related practice (NRP) among 1440 Polish students (aged 10–18 years) and identify the determining factors. Questions about NRK and NRP were thematically similar and referred to the recommendations of the Polish Pyramid of Healthy Nutrition and Lifestyle for Children and Adolescents (4–18 years). A cross-sectional study was conducted using the CAWI method. The respondents obtained an average of 51% of points in the NRK examination and 32% of points in the NRP examination. It was shown that NRP was positively associated with NRK (*p* < 0.001). The students’ NRK was positively determined by the following factors: being female (*p* < 0.001), older age of students (*p* < 0.001), living in a larger town (*p* = 0.012) and a higher level of education of the mother/legal guardian (*p* < 0.001). NRP was positively associated with greater physical activity of the students (*p* < 0.001). NRK and NRP were negatively associated with the subjects’ BMI (*p* = 0.029; *p* = 0.040, respectively). The analysis of NRK shows that the students’ knowledge regarding the consumption of milk, milk products and fish should be increased (17–20% correct answers). The analysis of NRP reveals that only 4–14% of students declared appropriate eating habits regarding the consumption of sweet and salty snacks, fish, wholegrain products and milk. This study highlights the need for targeted educational strategies to increase both the understanding and application of dietary guidelines among Polish adolescents for the prevention of diet-related diseases.

## 1. Introduction

Proper nutrition is one of the most important factors influencing the proper development of children and adolescents. It is also important for maintaining health in the future. Meanwhile, as children and adolescents become independent, the risk of consuming products rich in sugar, fat and salt, as well as deep-fried products, increases, while the consumption of products with high nutritional value is limited, which, combined with low physical activity, may lead to health disorders, including the development of obesity or diabetes [1,2]. According to the World Health Organization (WHO), over 390 million children and adolescents aged 5–19 were overweight in 2022. The prevalence of excess body weight among children and adolescents aged 5–19 years increased dramatically from 8% in 1990 to 20% in 2022 [3]. In Poland, excess body weight was found to occur in 23.6% of adolescents aged 10–19 years, including obesity in 7.2%. The numbers are constantly increasing [4].

In recent years, the topic of proper nutrition has been present in the public space in Poland, including schools. Classes devoted to this issue are included in the core curriculum of Polish schools [5]. Numerous educational programs concerning the principles of proper nutrition have been implemented in kindergartens and schools, including the “Keep balance” program financed by the Government of Switzerland and the Ministry of Health of the Republic of Poland, under which kindergartens/schools from all over the country that met specific requirements received diplomas for being “nutrition- and physical activity-friendly” [6]. A nationwide, multi-center “ABC of Healthy Eating” program was implemented in primary schools for children aged 11–13 [7], and the JEŻ program (i.e., Junior-Edu-Żywienie, meaning Junior-Edu-Nutrition) in grades I-VI of primary schools was implemented by the Warsaw University of Life Sciences (WULS) and the National Institute of Public Health–National Institute of Hygiene (NIPH-NIH) [8]. Also, the “Stay fit!” program has been implemented for years, addressing the students of grades V-VIII of primary schools and their parents and organized by the Chief Sanitary Inspectorate and the Polish Federation of Food Producers [9].

The Pyramid of Healthy Nutrition and Lifestyle for Children and Adolescents, along with 10 rules and comments, is an important tool that facilitates the transfer of information about proper nutrition. The Pyramid presents the principles of proper nutrition and the most important elements of healthy eating and lifestyle in a simple way. The implementation of the guidelines gives a chance for proper development and maintaining health in the future [10].

Professional recommendations on proper nutrition are widely available in Poland and should be known to children and adolescents. However, at the same time, the prevalence of excessive body weight is high in children and adolescents. In Poland, no research has assessed nutrition-related knowledge about proper nutrition expressed in the Pyramid of Healthy Nutrition and Lifestyle for Children and Adolescents in relation to nutrition-related practices while selecting factors determining them in a group of students aged 10–18.

Therefore, it was decided that it was necessary to assess whether information regarding nutritional knowledge was common and whether dietary recommendations were implemented by children and adolescents in practice, identifying nutritional irregularities and the factors determining them.

## 2. Materials and Methods

### 2.1. Study Participants

A cross-sectional study was conducted among Polish adolescents using the Computer-Assisted Web Interview (CAWI) method. The recruitment of students and schools was carried out using the nonprobability sampling method via social media (i.e., Facebook Inc.^©^ and Twitter). The survey was anonymous and voluntary. The inclusion criteria were the student’s consent, the consent of the parent/legal guardian to the student’s participation in the study, consent for the processing of personal data, the student’s age (10–18 years) and attendance at schools that agreed to conduct the survey. The students completed the survey using a Google Forms^©^ application. Each student completed the survey independently. The study was conducted during classes in computer labs. The survey provided the characteristics of food products. Only 12 survey questionnaires were rejected in the study (incomplete data).

The study was approved by the Bioethics Committee of the Institute of Human Nutrition Sciences of the Warsaw University of Life Sciences (No. 18/2021 of 1 June 2021) and was conducted in accordance with the Declaration of Helsinki.

### 2.2. Survey Study

An original questionnaire was prepared to assess nutrition-related knowledge (NRK) and nutrition-related practice (NRP). Questions about NRK and NRP were thematically similar and referred to the recommendations and principles of the Polish Pyramid of Healthy Nutrition and Lifestyle for Children and Adolescents (4–18 years) published by the National Institute of Public Health–National Institute of Hygiene (NIPH-NIH) in Warsaw [10]. The main part of the survey consisted of two thematic parts, with each part containing 12 questions related to NRK or NRP. In the final part of the survey, the students answered questions concerning sex, age, place of residence (village, town with up to 50,000 inhabitants or city with over 50,000 inhabitants), level of education of the mother/legal guardian (primary, secondary or higher) and level of physical activity (low—mainly sedentary in free time, moderate—half of the time sitting, half of the time active or high—spending most of the time actively). All questions were single-choice. The answers regarding knowledge included the option “I don’t know”. In addition, the survey included open-ended questions about the respondent’s height and weight (entered independently and verified by parents/guardians).

Before collecting data, a pilot study was conducted involving 30 students of different ages to ensure the transparency of the questions and their compliance with the recommendations of the Pyramid of Healthy Nutrition and Lifestyle for Children and Adolescents [10].

### 2.3. Assessment of Students’ NRK and NRP

The assessment of the students’ NRK and NRP was based on the points awarded to the students in the survey (Supplementary Materials, Tables S1 and S2). Each correct answer, in line with the recommendations of the Pyramid of Healthy Nutrition and Lifestyle for Children and Adolescents, was awarded 1 point, and 0 points were awarded for an incorrect answer or “I don’t know”. The maximum score for both the NRK and NRP indexes was 12.

### 2.4. Nutritional Status Assessment

The nutritional status of the students was assessed by calculating their body mass index (BMI) based on their self-reported weight and height. BMI values were compared to the BMI percentiles for age and sex in Poland to determine categories (underweight, normal weight, overweight, and obesity) in accordance with the guidelines [11].

### 2.5. Statistical Analysis

Statistical analysis was performed using the IBM SPSS Statistics29 software package (SPSS Inc., Chicago, IL, USA). The Shapiro–Wilk test was used to assess distributions. The data showed a skewed distribution. The results were expressed as the mean value, standard deviation, median, range, and percentage, depending on the type of variable. The NRK quartile distributions (on a scale of 0–12 points) were adopted as the differentiating factor in NRK and NRP assessments.

The Pearson’s Chi-square test (for categorical variables expressed as percentages) and the Kruskal–Wallis test (for continuous variables) were used to verify the significance between subgroups. Moreover, the Wilcoxon test was used to compare the results for dependent pairs (the NRK and NPR results on a scale of 0–12 points). Linear regression models were tested to assess the determinants of NRK and NRP by socio-demographic and lifestyle factors. Values of *p* ≤ 0.05 were considered statistically significant.

## 3. Results

### 3.1. Characteristics of the Group of Students

The study covered 1440 students, including 722 girls and 718 boys aged 10–18, living mostly in cities (65%) (Table 1). Compared to other quartiles (Q1–Q3), the quartile with the highest nutritional knowledge (Q4) encompassed the highest percentage of girls, students aged 13–15, students with a mother/legal guardian having completed higher education, normal BMI and moderate or high physical activity. Most students in quartile Q1 lived in rural areas.

### 3.2. Students’ NRK and NRP

It was found that the students’ NRK assessment differed significantly from their NRP assessment, both for all the respondents and in individual quartiles. The surveyed students obtained an average of 51% of points for correct answers in the NRK assessment (Q1: 27% and Q4: 73%) and only 32% of points for correct answers in the NRP assessment (Q1: 26% and Q4: 38%) (Table 2).

Significant differences were found in the responses regarding NRK depending on the knowledge quartile. The most correct answers were obtained to questions regarding knowledge about breakfast consumption (84%, Q1: 59% and Q4: 99%), sweet and salty snacks (73%, Q1: 40% and Q4: 96%; and 79%, Q1: 52% and Q4: 94%, respectively) and to the question “Eating which type of meat and/or meat products is the most recommended?” (75%, Q1: 54% and Q4: 87%). The fewest correct answers were given to the questions “How many servings of milk and/or dairy products should children and adolescents consume?” (17%, Q1: 8% and Q4: 36%) and “How often should children and adolescents eat sea fish?” (20%, Q1: 10% and Q4: 38%).

In the case of questions related to NRP, students gave the most correct answers to the question about the frequency of the consumption of lean meat and meat products (61%, Q1: 46% and Q4: 64%) and the level of physical activity (72%, Q1: 63% and Q4: 79%).

The fewest correct answers were obtained to questions concerning the frequency of the consumption of sweet and salty snacks (4%, Q1: 5% and Q4: 4%; and 6%, Q1: 6% and Q4: 7%, respectively), fish (8%, Q1: 9% and Q4: 10%), milk and wholegrain products (14%, Q1: 17% and Q4: 12%; and 14%, Q1: 10% and Q4: 17%, respectively). The students’ eating behavior related to the assessment of the consumption of milk and dairy products and fish, as well as sweet and salty snacks, did not correspond to the general knowledge of the respondents (with no statistically significant differences for the quartiles).

### 3.3. Socio-Demographic and Lifestyle Factors Determining NRK and NRP

It was estimated that the assessment of the students’ NRK was significantly and positively associated with age (the students’ knowledge increased with age), place of residence (larger residential centers) and higher education of the student’s mother/legal guardian (Table 3). Girls also had greater nutritional knowledge. In the case of BMI, a significant negative association was noted. A higher BMI was associated with poorer NRK results (*p* = 0.029).

In the assessment of factors determining the NRP of the respondents, a positive, statistically significant association was noted between the NRK assessment and physical activity (*p* < 0.0001). Similarly to the assessment of NRK, the association of the assessment of NRP with BMI was negative (*p* = 0.040). There was no impact from socio-demographic factors on the assessment of NRP among the students.

## 4. Discussion

The study confirms a statistically significant relationship between the students’ NRK and NRP results. Moreover, the authors show that the students’ NRK was associated with sex, age, place of residence, education of the student’s mother/legal guardian and BMI. In the case of the NRP assessment, an association was found between the students’ physical activity and their BMI. In addition, areas of knowledge necessary to be supplemented in educational remedial programs aimed at improving the eating habits of students aged 10–18 were identified.

### 4.1. The Assessment of Students’ Nutritional Knowledge and Eating Practices

In Poland, the Pyramid of Healthy Eating and Lifestyle for Children and Adolescents and the principles of healthy eating encompass the following recommendations [10]: the regular consumption of five meals (including breakfast); eating a variety of vegetables and fruits as often and as much as possible; the consumption of cereal products, especially whole grains; drinking at least 3–4 glasses of milk a day (they can be replaced with natural yogurt, kefir and, partially, cheese); the consumption of lean meats and the consumption of fish twice a week; choosing vegetable fats instead of animal fats; and drinking six glasses of water with meals and between meals. Additionally, it is recommended to avoid sweets (fruit or unsalted nuts and seeds are recommended instead), salty snacks and fast-food products. Daily physical activity is also recommended.

The obtained research results show that better implementation of dietary recommendations was accompanied by greater nutritional knowledge, although the levels differed significantly (with a poorer level of declared implementation). Other researchers assessing the knowledge and nutritional behavior of teenagers and students also reached similar conclusions [12,13,14,15,16]. However, not all studies in this area show such a relationship [17,18,19,20].

An interesting observation made in the context of the results obtained is that the vast majority of the surveyed youth knew some nutritional recommendations, e.g., regarding the consumption of breakfast or salty and sweet snacks and the type of meat more recommended for consumption, while only a low percentage of teenagers knew other equally important recommendations, such as those regarding the number of servings of milk or the frequency of fish consumption. No data are available to justify this phenomenon, but information about the health benefits of eating breakfast, as well as about the adverse effects of the excessive consumption of salty and sweet snacks, often repeated in various circumstances, reaches the awareness of young people. This may indicate the important role of repetition in education [21].

Young people most likely know that it is worth consuming dairy products and fish. However, our research shows that knowledge about how often or what number of portions should be consumed was insufficient. This indicates the need to change the teaching strategy. The lack of knowledge in this area may also result from the pyramidal form of the graphic, in which dairy products and fish are positioned in a way that may indicate limiting their consumption. If the pyramid is not studied along with the accompanying recommendations, the knowledge obtained may be incomplete or even incorrect. It may be worth developing a “healthy eating plate for children and adolescents”, similar to the recommendations for adults in Poland, which would be easier to understand for that group. However, to stay healthy, it is more important to follow dietary recommendations. Our research indicates a large discrepancy between knowledge and practice. Only 4% and 6% of respondents implemented rules regarding the consumption of sweet and salty snacks, respectively. The consumption of these products in excess promotes an excessive intake of sugar and salt, which are recognized as risk factors for the development of numerous metabolic diseases, including obesity, diabetes, cancer and cardiovascular diseases [22,23]. The higher BMI indices of the respondents who declared poorer implementation of dietary recommendations may only confirm such fears. In studies conducted in Spain, the authors noted that school-age children deviated from the principles of a health-promoting diet and increased the consumption of fast food and sweets despite knowledge about the harmfulness of those products. The authors stated it could also be due to skipping breakfast [24].

In the present study, we found that breakfast, the most important meal of the day, was regularly eaten only by half of the girls and boys. The results of the international research program “Health Behavior in School-aged Children” [25] and the Polish study “ABC of Healthy Eating” [26] also show that children and adolescents ate breakfast irregularly, although the change in this habit could be a strategy to maintain a correct BMI [27,28,29]. Moreover, the declared frequency of the consumption of grain products and fish and the number of milk servings was too low, which might have a negative correlation with the subjects’ BMI. It was confirmed that observing the correct frequency of the consumption of the above-mentioned product groups could promote a healthy body weight [30,31,32] and, in the case of fish, better academic results and grades in cognitive tests for teenagers [33,34]. It is worth incorporating this knowledge into educational programs addressed to students because research conducted in Austria shows that a higher level of nutritional knowledge was significantly associated with greater consumption of wholegrain bread and lower consumption of meat and energy drinks containing sweeteners [35].

### 4.2. Socio-Demographic and Lifestyle Factors Determining Students’ Knowledge and Implementation of Eating Habits

Being a female constituted a factor determining the students’ greater nutritional knowledge. The girls provided more correct answers in the knowledge test, which might be due to their greater interest in striving to maintain the desired figure. Studies previously conducted in Poland reveal that girls also achieved better results in the nutritional knowledge test, as well as in the assessment of the Pro-Healthy Diet Index, with a lower Non-Healthy Diet Index [7].

Chinese high school girls had better, although not statistically significant, knowledge about nutrition than boys, but boys achieved better results in terms of eating behaviors. However, the nutritional knowledge and eating behaviors of boys were not significantly correlated compared to girls. A positive correlation was found between nutritional knowledge and the regularity of eating breakfast both in girls and boys [36]. Iranian girls [37] were also characterized by greater nutritional knowledge than boys. Our previous research conducted as part of the Wise Nutrition–Healthy Generation program (WNHG, Warsaw, Poland) addressing secondary and high school students [38] demonstrated that boys were more likely to declare satisfaction with their body weight, which may also result in a lower need to expand nutritional knowledge. The relationship between NRK and body mass index (BMI) was also confirmed by other authors and showed that individuals who were dissatisfied with their body weight were less likely to follow dietary recommendations [38]. Therefore, it is worth looking for better methods of reaching out with knowledge about proper nutrition and its importance for proper development and health, especially to boys and people with abnormal BMI values. Our results also show that lower education levels of the mother/legal guardian were related to lower nutritional knowledge of the surveyed teenagers, which is confirmed in studies by other authors [39]. Nutritional knowledge varied depending on the age of the participants, particularly increasing in the older group [40]. A study conducted in Austria shows that the level of education and nutritional awareness of young people were influenced by a greater number of hours of nutritional education. Young people from rural areas were found to have better knowledge [35].

Research carried out among Italian teenagers aged 13–20 shows no relationship between nutritional knowledge and the respondents’ sex, BMI, physical activity and parents’ education. Nutritional knowledge scores varied significantly depending on the school district in which the youths were educated [41]. In the group of 12- to 16-year-old teenagers from Australia, a better level of the implementation of dietary recommendations was found in households with higher incomes. It was also found that a greater number of hours devoted by parents/guardians to professional work had a negative impact on the implementation of this type of recommendation. The calculated average diet index for children and teenagers was low and did not differ, as in our study, depending on sex [42]. However, at the same time, it was significantly poorer in adolescents aged 12–16 compared to the results of children from the 4–7 and 8–11 age groups [42].

In our research, demographic factors (i.e., sex, age, place of residence, and education level of the mother/legal guardian) did not determine the implementation of dietary recommendations, which requires further research in the future. However, Iranian studies conducted on adolescents aged 10–19 show higher rates of the implementation of dietary recommendations by boys, younger children and children living in cities [39]. The relationship between socio-demographic and lifestyle factors (age, sex, parents’ education level, and income level) with dietary practices is also confirmed by other authors [15,16,17,43,44,45,46]. It was also shown that the implementation of dietary recommendations was weakly related to BMI or the z-score in the waist circumference of adolescents aged 12–16 [42].

### 4.3. Strengths and Limitations of the Study

Our study provides important observations regarding students’ NRK and NRP, allowing for their comprehensive assessment in relation to the Polish recommendations of the Pyramid of Healthy Nutrition and Lifestyle for Children and Adolescents. Our research highlights a selection of areas of knowledge that require supplementation in educational compensatory programs, as well as the identification of areas that require changes based on the respondents’ NRP. Moreover, this study identifies the determinants of NRK and NPR, which may contribute to understanding which groups of students require more attention and personalized efforts to improve NRK and NPR in the future. This study fills the gaps in the above areas.

Our study has several strengths. This is a pioneering study in Poland, reflecting nutritional recommendations in questions about knowledge and their application in practice (with analogous knowledge/implementation questions). A valuable result of the conducted research includes the precise identification of areas of knowledge and declared practice that require supplementation/correction. The study was conducted in a large group (N = 1440) and wide age range (10–18 years) of Polish adolescents, taking account of various socio-demographic factors (age, sex, place of residence, and education level of mother/legal guardian), physical activity and the students’ BMI. The authors did not find any studies assessing the relationship between nutrition-related practice and physical activity, which may indicate that our study is probably the first in this area. This approach is extremely important in preparing personalized educational programs aimed at improving BMI and health.

This study also has limitations. The survey was not verified for reliability. However, before collecting data, a pilot study was conducted, including 30 students of different ages, to ensure the clarity of the questions and their compliance with the recommendations of the Pyramid of Healthy Nutrition and Lifestyle for Children and Adolescents. A larger percentage of adolescents aged 16–18 participated in the study compared to teenagers from other age groups, which may be due to the greater knowledge of computer techniques in this group. (The study was conducted using the CAWI method.) Moreover, nonprobability sampling was used when selecting the study group, and each BMI was calculated based on data declared by the students, which could have influenced the assessment of the incidence of obesity. The study allowed for the identification of factors determining NRK and NRP, but as a cross-sectional study, it did not allow for establishing causal relationships.

## 5. Conclusions

It was confirmed that the implementation of nutritional recommendations in practice was associated with the students’ nutritional knowledge, with a much lower level of nutritional practice. Nutrition-related knowledge was positively associated with being female, older student age, living in a larger city and higher education of the mother/legal guardian and negatively associated with the BMI. The implementation of dietary recommendations was positively associated with the greater physical activity of the students and negatively with lower BMI. It was found that, in the future, educational programs for Polish adolescents should be supplemented with knowledge regarding the frequency of the consumption of milk and milk products, wholegrain products and fish, as well as limiting the consumption of sweet and salty snacks. This study provides arguments for nutrition and dietetics professionals to develop and implement targeted, personalized nutrition strategies for students aged 10–18 years, with the aim of improving their nutritional status and reducing the risk of developing nutritional diseases in the future.

## Figures and Tables

**Table 1:** Characteristics of the studied group of students according to NRK quartiles (N = 1440).

Factor	Categories	Total N = 1440	NRK Q1–Q4 *				Chi2 Test
			Q1 N = 360	Q2 N = 360	Q3 N = 360	Q4 N = 360	
Sex (%)	girls	50	40	45	54	61	<0.001
	boys	50	60	55	46	39	
Age (years)	x ± sd Me min–max	14.4 ± 2.6 15.0 10–18	13.3 ± 2.7 13.0 10–18	14.7 ± 2.6 15.0 10–18	15.2 ± 2.7 16.0 10–18	14.4 ± 2.2 15.0 10–18	<0.001
Age (%)	10–12 years	30	46	23	27	24	<0.001 **
	13–15 years	28	29	29	11	44	
	16–18 years	42	25	48	62	32	
Place of residence (%)	village	35	42	35	26	39	<0.001
	town < 50,000	19	25	17	16	18	
	city > 50,000	46	33	48	59	43	
Education level of mother/legal guardian (%)	primary	12	22	12	6	6	<0.001
	secondary	32	33	37	33	26	
	higher	56	46	51	61	68	
Physical activity (%)	low	18	22	21	21	10	0.008
	moderate	66	61	62	68	72	
	high	12	13	11	8	17	
BMI (%)	underweight	11	9	12	12	9	0.012
	normal	71	66	68	73	76	
	overweight	17	23	19	13	13	
	obesity	2	3	1	1	2	

sd, standard deviation, Me, median, * Scale: 0–12 points, ** The Kruskal–Wallis test.

**Table 2:** Assessment of students’ NRK and NRP according to NRK quartiles (N = 1440) (%).

Questions	Total N = 1440	NRK Q1–Q4 *				Chi2 Test
		Q1 N = 360	Q2 N = 360	Q3 N = 360	Q4 N = 360	
NRK (% of correct answers)						
How many meals should children and adolescents eat a day?	58	24	45	75	86	<0.001
Which meal eaten regularly is particularly important for well-being at school?	84	59	84	95	99	<0.001
How often should children and adolescents eat fruit and vegetables?	57	24	49	66	89	<0.001
Which products contain more dietary fiber?	61	26	51	77	92	<0.001
How many servings of milk and/or dairy products should children and adolescents consume a day?	17	8	12	11	36	<0.001
Which type of meat and/or meat products is most recommended for consumption?	75	54	74	86	87	<0.001
How often should children and adolescents eat sea fish?	20	10	16	16	38	<0.001
What is at the top of the Healthy Nutrition and Lifestyle Pyramid?	32	9	15	24	81	<0.001
Which products should replace sweets?	73	40	67	89	96	<0.001
Which products contain a lot of salt?	79	52	82	89	94	<0.001
What is the recommended amount of water consumption for children and adolescents?	58	24	53	77	79	<0.001
What is at the base of the Healthy Nutrition and Lifestyle Pyramid?	39	15	29	40	73	<0.001
x ± sd * Me min–max NRK % of correct answers	6.1 ± 2.2 6.0 0–11 51%	3.3 ± 1.2 4.0 0–5 27%	5.5 ± 0.8 5.0 4–7 46%	7.1 ± 0.7 7.0 6–8 59%	8.8 ± 1.0 9.0 7–11 73%	<0.001 **
NRP (% of correct answers)						
How many meals a day do you usually eat?	28	20	24	29	38	<0.001
Do you eat breakfast in the morning before going to school?	49	41	48	55	54	<0.001
How often do you eat fruit and vegetables?	31	13	25	32	54	<0.001
How often do you eat whole grain products?	14	10	10	19	17	<0.001
How many servings of milk and/or dairy products do you consume per day?	14	17	13	13	12	0.292
Which type of meat and/or meat products do you eat most often?	61	46	59	74	64	<0.001
How often do you eat fish?	8	9	5	7	10	0.067
What fat are the foods you eat fried in?	56	41	54	63	67	<0.001
How often do you eat sweets?	4	5	3	4	4	0.261
How often do you eat salty snacks?	6	6	6	6	7	0.893
How much water do you drink a day?	45	37	46	48	48	0.010
What is your physical activity?	72	63	69	79	79	<0.001
x ± sd * Me min–max NRP % of correct answers	3.9 ± 1.7 4.0 0–9 32%	3.1 ± 1.5 3.0 0–7 26%	3.6 ± 1.7 3.0 0–9 30%	4.3 ± 1.5 4.0 1–9 36%	4.5 ± 1.7 4.0 0–9 38%	<0.001 **
NRK and NRP comparison *	<0.001 ***	0.005 ***	<0.001 ***	<0.001 ***	<0.001 ***	

sd, standard deviation, Me, median, * Scale: 0–12 points, ** Kruskal–Wallis test, *** Wilcoxon test.

**Table 3:** Association of socio-demographic and lifestyle factors with the students’ NRK and NRP (N = 1440).

Factor	Unstandardized B (95% CI)	Standardizedβ	*p*-Value
NRK *			
Sex	−0.670 (−0.891; −0.449)	−0.151	<0.001
Age	0.144 (0.100; 0.188)	0.171	<0.001
Place of residence	0.164 (0.036; 0.292)	0.066	0.012
Education level of mother/legal guardian	0.648 (0.489; 0.807)	0.202	<0.001
BMI (categories)	−0.213 (−0.404; −0.022)	−0.055	0.029
NRP *			
Sex	−0.148 (−0.306; 0.010)	−0.044	0.067
Age	−0.025 (−0.056; 0.007)	−0.039	0.121
Place of residence	−0.032 (−0.121; 0.058)	−0.017	0.486
Education level of the mother/legal guardian	0.045 (−0.068; 0.158)	0.018	0.433
Level of physical activity	0.896 (0.789; 1.002)	0.382	<0.001
BMI (categories)	−0.140 (−0.273; −0.007)	−0.048	0.040
NRK *	0.253 (0.217; 0.289)	0.330	<0.001

* Scale: 0–12 points; NRK: linear regression, adjusted R2 = 0.126, F = 42.591, *p* < 0.001; NRP: linear regression, adjusted R2 = 0.280, F = 80.779, *p* < 0.001.

## Data Availability

The dataset used and/or analyzed during this study is available from the corresponding author upon reasonable request.

## Supplementary Materials

The following supporting information can be downloaded at: https://www.mdpi.com/article/10.3390/nu16111611/s1. Table S1: List of questionnaire items measuring nutrition-related knowledge (NRK) in accordance with national nutritional recommendations for children and adolescents [10] with rewarded response; Table S2: List of questionnaire items measuring nutrition-related practice (NRP) in accordance with national nutritional recommendations for children and adolescents [10] with rewarded response.
