# Supplementary material for: Nutrition-Related Knowledge and Nutrition-Related Practice among Polish Adolescents—A Cross-Sectional Study
Source: Nutrients. 2024 May 25;16(11):1611. doi: 10.3390/nu16111611 (PMC11175151; doi:10.3390/nu16111611)
Supplement: Supplementary file 1 [file nutrients-16-01611-s001.zip › nutrients-3017622-supplementary.pdf]

Supplementary Materials

Table S1. List of questionnaire items measuring nutrition-related knowledge (NRK) in accordance with national nutritional recommendations for children and adolescents [10] with rewarded response.

No	Questions	Awarded answer (1 point)
Q1	How many meals should children and adolescents eat a day?	5 meals
Q2	Which meal eaten regularly is particularly important for well-being at school?	breakfast
Q3	How often should children and adolescents eat fruit and vegetables?	few times a day
Q4	Which products contain more dietary fiber?	wholegrain dark cereal products, e.g. wholemeal bread, brown rice, wholegrain pasta
Q5	How many servings of milk and/or dairy products should children and adolescents consume a day?	at least 3-4 servings a day
Q6	Which type of meat and/or meat products is most recommended for consumption?	lean meat, e.g. chicken/turkey breast, baked or boiled pork loin
Q7	How often should children and adolescents eat sea fish?	at least 2 servings a week
Q8	What is at the top of the Healthy Nutrition and Lifestyle Pyramid?	oils and nuts
Q9	Which products should replace sweets?	fruit and nuts
Q10	Which products contain a lot of salt?	fast food
Q11	What is the recommended amount of water consumption for children and adolescents?	6 glasses a day
Q12	What is at the base of the Healthy Nutrition and Lifestyle Pyramid?	physical activity

Table S2. List of questionnaire items measuring nutrition-related practice (NRP) in accordance with national nutritional recommendations for children and adolescents [10] with rewarded response.

No	Questions	Awarded answer (1 point)
Q1	How many meals a day do you usually eat?	5 meals
Q2	Do you eat breakfast in the morning before going to school?	yes
Q3	How often do you eat fruit and vegetables?	few times a day
Q4	How often do you eat whole grain products?	few times a day
Q5	How many servings of milk and/or dairy products do you consume per day?	at least 3-4 servings a day
Q6	Which type of meat and/or meat products do you eat most often?	lean meat, e.g. chicken/turkey breast, baked or boiled pork loin
Q7	How often do you eat fish?	few times a week
Q8	What fat are the foods you eat fried in?	refined rapeseed oil, olive oil
Q9	How often do you eat sweets?	never or less than 1-3 times a month
Q10	How often do you eat salty snacks?	never or less than 1-3 times a month
Q11	How much water do you drink a day?	6-8 glasses a day
Q12	What is your physical activity?	moderate – half of the time sitting, half of the time active; high – spending most of the time actively
